# Development of a *Drosophila* Cell-Based Error Correction Assay

**DOI:** 10.3389/fonc.2013.00187

**Published:** 2013-07-23

**Authors:** Jeffrey D. Salemi, Philip T. McGilvray, Thomas J. Maresca

**Affiliations:** ^1^Biology Department, University of Massachusetts, Amherst, MA, USA; ^2^Molecular and Cellular Biology Graduate Program, University of Massachusetts, Amherst, MA, USA

**Keywords:** error correction, kinesin-5, aurora B kinase, kinetochore, spindle, *Drosophila*

## Abstract

Accurate transmission of the genome through cell division requires microtubules from opposing spindle poles to interact with protein super-structures called kinetochores that assemble on each sister chromatid. Most kinetochores establish erroneous attachments that are destabilized through a process called error correction. Failure to correct improper kinetochore-microtubule (kt-MT) interactions before anaphase onset results in chromosomal instability (CIN), which has been implicated in tumorigenesis and tumor adaptation. Thus, it is important to characterize the molecular basis of error correction to better comprehend how CIN occurs and how it can be modulated. An error correction assay has been previously developed in cultured mammalian cells in which incorrect kt-MT attachments are created through the induction of monopolar spindle assembly via chemical inhibition of kinesin-5. Error correction is then monitored following inhibitor wash out. Implementing the error correction assay in *Drosophila melanogaster* S2 cells would be valuable because kt-MT attachments are easily visualized and the cells are highly amenable to RNAi and high-throughput screening. However, *Drosophila* kinesin-5 (Klp61F) is unaffected by available small molecule inhibitors. To overcome this limitation, we have rendered S2 cells susceptible to kinesin-5 inhibitors by functionally replacing Klp61F with human kinesin-5 (Eg5). Eg5 expression rescued the assembly of monopolar spindles typically caused by Klp61F depletion. Eg5-mediated bipoles collapsed into monopoles due, in part, to kinesin-14 (Ncd) activity when treated with the kinesin-5 inhibitor *S*-trityl-L-cysteine (STLC). Furthermore, bipolar spindles reassembled and error correction was observed after STLC wash out. Importantly, error correction in Eg5-expressing S2 cells was dependent on the well-established error correction kinase Aurora B. This system provides a powerful new cell-based platform for studying error correction and CIN.

## Introduction

During cell division, correct segregation of the genome requires that replicated chromosomes become bioriented, with each sister chromatid attached to dynamic microtubules from opposite spindle poles. The interaction between chromosomes and microtubules is mediated by a protein complex, called the kinetochore, which assembles on the centromeres of each sister chromatid as the cell prepares to divide. The kinetochore is comprised of ∼100 known proteins that are present in multiple copies and organized into a conserved molecular architecture consisting of two spatially distinct electron-dense regions generally referred to as the inner and outer kinetochore (1–5). Kinetochore-microtubule (kt-MT) interactions are mediated through numerous outer kinetochore factors. The establishment and maintenance of end-on kt-MT attachments requires the KMN (KNL1/Blinkin, Mis12 complex, Ndc80 complex) network while mature end-on attachments are reinforced through recruitment of the microtubule-binding Ska1 complex to bioriented kinetochores (6–10).

Biorientation is the ideal chromosomal configuration but it is by no means a default state. In fact, a vast majority of chromosomes in mammalian oocytes establish multiple incorrect kt-MT interactions before achieving biorientation (11). Cells have evolved an essential network of regulatory components that correct flawed interactions between kinetochores and spindle microtubules. The process by which improper kt-MT attachments are selectively destabilized is called error correction. The best characterized error correction regulator is Aurora B kinase (ABK) (12). Prior to anaphase, ABK is highly enriched in the centromeric chromatin that underlies the inner kinetochore. The microtubule-binding activities of both the KMN network and the Ska1 complex are negatively regulated by ABK-mediated phosphorylation (13–17). At improperly attached kinetochores, it is postulated that attachment factors are positioned close enough to ABK to become phosphorylated thereby reducing their affinity for microtubules (16, 18). ABK inhibition leads to a prevalence of incorrect interactions called syntelic attachments where each sister kinetochore is attached to the same spindle pole (19–21). Therefore, erroneous kt-MT interactions are commonplace but typically corrected by ABK.

The ability to visualize the conversion of erroneous kt-MT attachments to bioriented attachments in living cells would provide valuable insight into the error correction process. Indeed, a live-cell error correction assay was previously developed in mammalian cells (21). In the established assay, incorrect kt-MT attachments are induced by chemical inhibition of mammalian kinesin-5 (Eg5). Since Eg5 pushes spindle poles apart, inhibiting kinesin-5 motor activity leads to monopolar spindle assembly and results in many sister kinetochores becoming syntelically attached to the single spindle pole. Small molecule inhibitors of Eg5 can be “washed-out” to reverse their effects and during the subsequent reorganization from monopole to bipole incorrect attachments are corrected in an ABK-dependent manner.

*Drosophila melanogaster* S2 cells have proven to be an excellent model for studying mitotic spindle assembly and kinetochore function. The fact that *Drosophila* have only four chromosomes makes visualization of kt-MT attachments considerably easier than in other model cell types such as HeLa that have>100 kinetochores. Although S2 cell lines are not typically diploid (22) and tend to become tetraploid through passaging (23), the number of chromosomes when compared to HeLa cells still yields distinct advantages for visualization. Furthermore, S2 cells are exceedingly amenable to high-throughput whole-genome RNAi screening (24, 25). Unfortunately, the error correction assay cannot be applied to S2 cells because Klp61F (*D. melanogaster* kinesin-5) is unaffected by kinesin-5 inhibitors such as monastrol and *S*-trityl-l-cysteine (STLC) (26–28). Rendering *Drosophila* S2 cells susceptible to Eg5 inhibitors would overcome these limitations and provide a new cell-based tool for examining error correction and CIN.

## Materials and Methods

### Cell culture

*Drosophila* S2 cells were cultured at 24°C in Schneider’s media (Life Technologies) supplemented with 10% heat inactivated fetal bovine serum (Life Technologies) and 0.5× antibiotic-antimycotic cocktail (Life Technologies).

### Generation of S2 cell lines

The *Homo sapiens* Eg5 (kinesin-5) gene was amplified from pCS2-EGFP-Eg5 (gift of Patricia Wadsworth, UMASS, Amherst) with a 5^′^ *Kpn*I site and 3^′^ *Spe*I site. The resulting product was inserted into the multiple cloning site of a pMT/V5 His-B vector (Invitrogen) containing the mCherry gene and Blasticidin resistance marker. Human Eg5-mCherry DNA was transfected into stable Hygromycin B resistant, GFP-α-tubulin-expressing S2 cells using the Effectene Transfection Reagent system (Qiagen), according to product directions. The transfected cells were grown in Schneider’s media (Invitrogen) containing 10% Fetal Bovine Serum (Invitrogen). After 4 days, they were transferred to a 25 cm^2^ flask. Cells were then grown in media containing Blasticidin S HCL (Invitrogen) at a concentration of 0.025 mg/ml until cell death ceased. At that point cells were maintained in media containing no Blasticidin. Eg5-mCherry was induced by adding 500 μM CuSO_4_ to the media 6–18 h before an experiment.

### Production of dsRNAs

DNA templates for Klp61F (CG9191), Ncd (CG7831), and Aurora B (CG6620) were produced to contain ∼500 bp of complementary sequence flanked with the T7 promoter sequence (Table S1 in Supplementary Material). dsRNAs were synthesized overnight at 37°C from the DNA templates using the T7 RiboMax™ Express Large Scale RNA Production System (Promega). RNAi experiments were done as previously described (29).

### Live-cell imaging

Cells were seeded onto Concanavalin A (Sigma) treated acid-washed coverslips (Corning) for 1 h. The coverslips were assembled into rose chambers containing Schneider’s media and imaged at room temperature. Cells were imaged on a Nikon TiE inverted microscope with a cooled CCD Orca R2 camera (Hamamatsu) using Nikon 100 × 1.4 NA Plan Apo violet corrected (VC) series differential interference contrast (DIC) and 40 × 1.3 NA Plan Fluor DIC objectives. Metamorph software (Molecular Devices) was used to control the imaging system as well as for image processing and analysis.

### Immunofluorescence

S2 cells were allowed to adhere to ConA-coated cover slips before being quickly rinsed in BRB80 and then fixed for 10 min in 10% paraformaldehyde. Cells were then permeabilized for 8 min in 1× PBS + 1% Triton, washed 3× in 1× PBS + 0.1% Triton, and blocked for at least 30 min in Boiled Donkey Serum. Primary antibodies were diluted into Boiled Donkey Serum: anti-phosphoH3 Serine 10 (Abcam) at 1:20,000, DM1α (anti-α Tubulin antibody, Sigma-Aldrich) at 1:1000, and anti-Ndc80 (generated by Thomas J. Maresca) at 1:100. All appropriate secondary antibodies (Jackson ImmunoResearch) were diluted 1:200 in Boiled Donkey Serum. Cells were treated with DAPI (1:100) and sealed in mounting media containing 20 mM Tris (pH8.0), 0.5% *N*-propyl gallate, and 90% glycerol.

### Western blotting

A total of 10 μg of protein was loaded into a 10% SDS-PAGE gel, run out, and transferred to PVDF membrane (BioRad) in transfer buffer containing 10% methanol. All antibodies were diluted in TBS with 0.1% Tween and 5% milk. The membrane was first incubated with anti-Klp61F serum (gift of Jonathan Scholey, UC, Davis) at 1:500 and DM1α (anti-α Tubulin antibody) (Sigma-Aldrich) at 1:1000 and then probed with anti-Human polyclonal Eg5 antibody (Novus Biologicals, gift of Patricia Wadsworth, UMASS Amherst) at 1:500. Rabbit and mouse HRP secondary antibodies (Jackson ImmunoResearch) were used in conjunction with their respective primaries and imaged with a GBox system controlled by GeneSnap software (Syngene).

## Results

### Human Eg5 localizes normally throughout the cell cycle in *Drosophila* S2 cells

**Figure 1:**
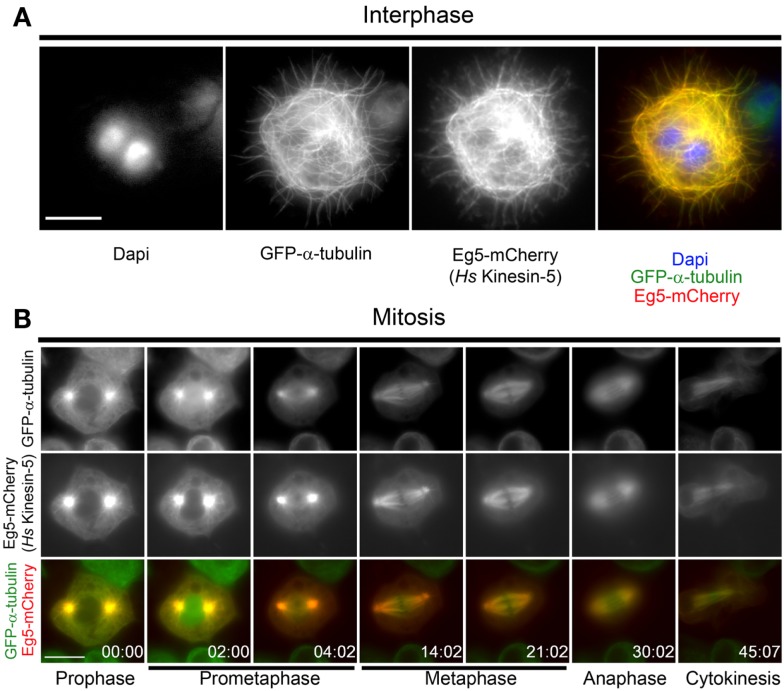
**Localization of human Eg5 in *Drosophila melanogaster* S2 cells**. **(A)** Whole cell two-color image of a GFP-α-tubulin (green), Eg5-mCherry (red) expressing *Drosophila* S2 cell line. Interphase localization of human Eg5 to microtubules is evident. **(B)** Still frames from a time-lapse of a mitotic cell expressing GFP-α-tubulin (green), Eg5-mCherry (red). In mitosis Eg5 localizes to spindle microtubules and is enriched at spindle poles. Scale bars are 10 μm.

To investigate the behavior of human Eg5 (*Hs*-Kinesin-5) in *Drosophila*, a stable *Drosophila* S2 cell line expressing GFP-α-tubulin and Eg5-mCherry under the control of a copper-inducible promoter was generated. During interphase, Eg5 is excluded from the nucleus and associates with microtubules (30) while in mitosis the motor localizes to spindle microtubules and is enriched at spindle poles (31). Eg5 expression was induced overnight with copper sulfate and mCherry fluorescence was visualized in both live and fixed cells. Exogenous Eg5 associated with microtubules in interphase cells (Figure 1A). While the interphase microtubule localization of human Eg5 in S2 cells could be attributed, in part, to Eg5 over-expression, it may also reflect an under-appreciated role for Eg5, which is assumed to be a mitotic kinesin, in interphase. In mitosis, Eg5 became highly enriched at centrosomes in prophase and remained associated with the centrosomes through the entirety of mitosis (Figure 1B; Movie S1 in Supplementary Material). Following nuclear envelope breakdown in prometaphase, Eg5-mCherry associated with the microtubules of the assembling spindle and by metaphase Eg5 was enriched near the spindle poles. Eg5 remained on midzone microtubules through anaphase and localized at the midbody during cytokinesis (Figure 1B; Movies S1 and S2 in Supplementary Material). Since Eg5-mCherry localized normally in both interphase and mitotic *Drosophila* S2 cells, the functionality of the motor in S2 cells was next investigated.

### Human Eg5 rescues knockdown of *Drosophila* kinesin-5

**Figure 2:**
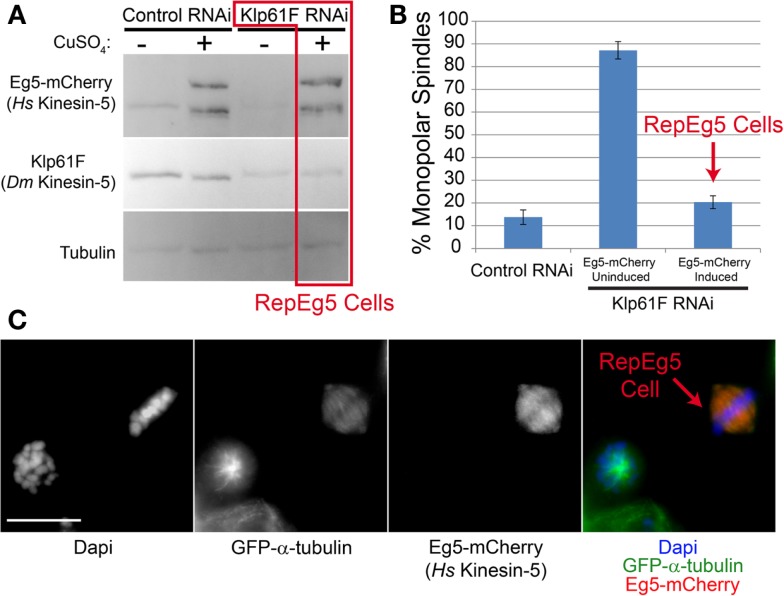
**Human Eg5 rescues depletion of the *Drosophila* kinesin-5 family member Klp61F**. **(A)** Western blot analysis of cell lysates prepared from induced and uninduced; control and Klp61F RNAi-treated cells blotted with anti-Eg5 to monitor induction of Eg5-mCherry, anti-Klp61F to confirm depletion of the motor and anti-α-tubulin as a loading control. Cells depleted of Klp61F and with Eg5-mCherry induced are referred to as RepEg5 cells. **(B)** Quantification of the percentage of cells with monopolar spindles in control RNAi cells versus Klp61F-depleted Eg5-mCherry induced and uninduced cells. **(C)** An example of a field of view containing a RepEg5 cell with a bipolar spindle next to a Klp61F-depleted cell that is not expressing Eg5-mCherry and has a monopolar spindle. In the merged image GFP-α-tubulin is shown in green, Eg5-mCherry in red, and DAPI-stained DNA in blue. Error bars are standard error of the mean (SEM). Scale bar is 10 μm.

Klp61F is the *Drosophila* kinesin-5 family member and homolog of Eg5 (32). While Klp61F exhibits significant homology to human Eg5 in its motor domain, the remainder of the protein is highly divergent and; interestingly, Klp61F is not sensitive to chemical inhibitors that disrupt Eg5 function across a range of species (26– 28). Furthermore, to our knowledge, the ability of human Eg5 to complement Klp61F function has never been investigated. Thus, the capacity of Eg5-mCherry to rescue Klp61F depletion in S2 cells was tested. In the absence of Klp61F, *Drosophila* cells fail to assemble bipolar spindles and instead generate monopoles (33, 34). The same was true in uninduced Eg5-mCherry expressing cells as ∼80% of mitotic cells assembled monopoles following depletion of Klp61F by RNAi (Figures 2A,B). To the contrary, nearly ∼90% of mitotic cells expressing Eg5-mCherry assembled bipolar spindles following induction (Figure 2B). After induction, Eg5-mCherry expression, which was confirmed by western blot analysis (Figure 2A), varied on a cell-by-cell basis with some cells exhibiting undetectable mCherry fluorescence. The variation in expression levels served as an internal control given that Klp61F-depleted cells without Eg5 assembled monopoles while nearby Eg5-mCherry-expressing cells nearly always built bipolar spindles (Figure 2C). Thus, Eg5-mCherry rescues Klp61F depletion in *Drosophila* S2 cells. Henceforth, Klp61F-depleted cells that express detectable levels of Eg5-mCherry will be referred to as Replacement Eg5 (RepEg5) cells.

### RepEg5 cells are sensitive to chemical inhibition of Eg5

**Figure 3:**
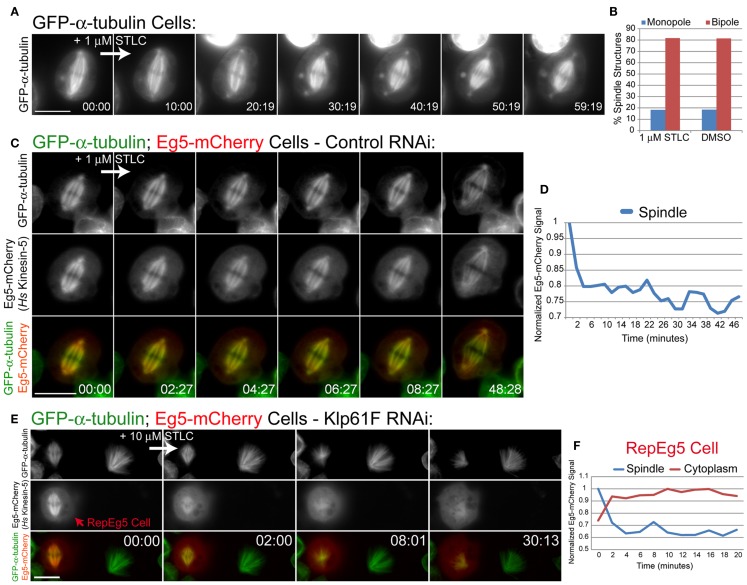
**RepEg5 cells are rendered sensitive to chemical inhibition of Eg5**. **(A)** Still frames from a time-lapse of an MG132-treated GFP-α-tubulin-expressing cell before and after addition of 1 μM STLC. **(B)** Quantification of spindle structures in S2 cells treated overnight with either 1 μM STLC or 1% DMSO. Spindle morphologies are identical after each treatment (*n* = 104 structures for STLC; *n* = 108 structures for DMSO). **(C)** Still frames from a time-lapse of an MG132-treated Eg5-mCherry-expressing cell before and after addition of 1 μM STLC. Introduction of STLC does not cause spindle collapse despite the fact that spindle-associated levels of Eg5-mCherry decrease. **(D)** Quantification of spindle-associated Eg5-mCherry fluorescence intensity normalized against GFP-α-tubulin signal. Spindle-associated Eg5-mCherry levels drop by 20% following the addition of 1 μM STLC. **(E)** Still frames from a time-lapse of a RepEg5 cell with a bipolar spindle next to a Klp61F-depleted cell that is not expressing Eg5-mCherry and has a monopolar spindle. The bipolar spindle in the RepEg5 cell collapses into a monopole within 8 min of introducing 10 μm STLC, accompanied by reduction in spindle-associated Eg5-mCherry and a concomitant increase in cytoplasmic mCherry fluorescence. **(F)** Quantification of Eg5-mCherry intensity on the spindle (blue trace), which decreases by 40% within ∼4 min of adding STLC, and the cytoplasm (red trace) in the RepEg5 cell shown in E. In the merged images GFP-α-tubulin is shown in green and Eg5-mCherry is shown in red. Scale bars are 10 μm.

Chemical inhibition of Eg5 results in monopolar spindle assembly (35). It has been reported that the widely used small molecule Eg5 inhibitors STLC and monastrol do not affect Klp61F (26, 28). Accordingly, treatment of GFP-α-tubulin-expressing cells with the inhibitor *S*-trityl-l-cysteine (STLC) did not cause spindle collapse or assembly of monopoles (Figure 3A; Movie S3 in Supplementary Material). Furthermore, spindle morphologies in cells subjected to overnight STLC treatment were identical to those in cells treated overnight with DMSO (Figure 3B). Spindle morphologies were analyzed by observing the organization of the chromosomes in mitotic cells as well as spindle pole positioning. Cells containing spindle poles that were within several microns of each other and that had splayed chromosomes arranged at the periphery of the microtubule array were deemed to have monopolar spindle. Cells with clearly separated spindle poles located on either side of a linear arrangement of chromosomes were considered to possess bipolar spindles. These data further demonstrate that STLC does not inhibit Klp61F activity.

To determine whether S2 cells were permeable to STLC, spindle-associated Eg5-mCherry fluorescence was measured following addition of the inhibitor to control RNAi-treated cells. STLC inhibits the microtubule-stimulated ATPase activity of Eg5 and causes spindle-associated Eg5 levels to drop and become diffusive in cells (36, 37). Similarly, addition of 1 μM STLC to Eg5-expressing S2 cells resulted in a 20% decrease in Eg5-mCherry spindle fluorescence within 2 min of introducing the drug (Figures 3C,D; Movie S4 in Supplementary Material). The observed reduction in mCherry fluorescence revealed that STLC can rapidly cross the plasma membrane in S2 cells and that STLC treatment causes a fraction of Eg5 to dissociate from spindle microtubules even when the motor is expressed in *Drosophila* (Figure 3D). Despite the fact that the exogenous Eg5-mCherry was evidently targeted by STLC, the presence of endogenous Klp61F prevented STLC-induced spindle collapse in control RNAi cells (Figure 3C; Movie S4 in Supplementary Material). The effects of STLC in Eg5-mCherry expressing cells following depletion of Klp61F by RNAi was investigated next. As previously discussed, RepEg5 cells assembled bipolar spindles in the absence of Klp61F (Figures 2C and 3E). Importantly, bipolar spindles in RepEg5 cells collapsed into monopoles within 10 min of introducing STLC (Figure 3E; Movie S5 in Supplementary Material). Spindle-associated and cytoplasmic Eg5-mCherry fluorescence intensities were also quantified during STLC treatment of RepEg5 cells. In the example shown in Figure 3E; Movie S5 in Supplementary Material, as the bipole collapsed, there was a 40% reduction in spindle-associated Eg5 and a concomitant increase in cytoplasmic fluorescence within 10 min of introducing STLC (Figure 3F). Thus, RepEg5 S2 cells were successfully rendered sensitive to chemical inhibition by STLC.

### STLC-induced spindle collapse in RepEg5 cells requires the minus end-directed *Drosophila* kinesin-14 motor Ncd

**Figure 4:**
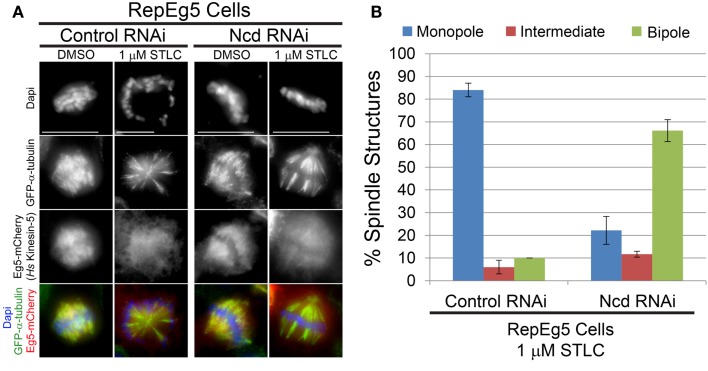
**Efficient collapse of bipolar spindles into monopoles in STLC-treated RepEg5 cells requires the minus end-directed *Drosophila* kinesin-14 motor Ncd**. **(A)** Representative maximum projection images of spindle morphologies in control RNAi- and Ncd RNAi-treated RepEg5 cells in either DMSO or 1 μM STLC. In the merged images GFP-α-tubulin is green, Eg5-mCherry is red, and DAPI-stained DNA is blue. **(B)** Quantification of spindle structures in RepEg5 cells treated with 1 μM STLC in the presence and absence of Ncd (*n* = 200 spindles in control RNAi; *n* = 206 structures in Ncd RNAi). Error bars are SEM. Scale bars are 10 μm.

Spindle collapse following kinesin-5 inhibition is mediated by opposing forces that are generated by minus end-directed motors (56). Dynein is the principal minus end-directed motor opposing Eg5 in vertebrates (39–42) while the motor Ncd (kinesin-14) fills this role in *Drosophila* (29, 33, 34, 38, 43). The contribution of Ncd to the STLC-induced spindle collapse seen in RepEg5 cells was; therefore, investigated. In agreement with previous observations in *Drosophila* cells (34, 44, 45), Ncd knockdown resulted in the assembly of spindles with unfocused poles (Figure 4A). Addition of STLC to control RNAi-treated cells caused dissociation of Eg5 from spindle microtubules and monopolar spindle assembly in more than 80% of RepEg5 cells (Figures 4A,B). There was a fourfold reduction in the percentage of RepEg5 cells with monopolar spindles following STLC treatment in Ncd-depleted cells. In fact, 66% of the Ncd-depleted RepEg5 cells assembled bipolar spindles with unfocused poles in the presence of STLC. To be confident that the cells analyzed in these experiments were indeed STLC-inhibited, only cells with high levels of diffuse cytoplasmic Eg5 were scored for spindle morphology. Thus, Ncd activity contributes to collapsing bipoles into monopoles in STLC-treated RepEg5 cells.

### Bipolar spindles reassemble following STLC wash out

**Figure 5:**
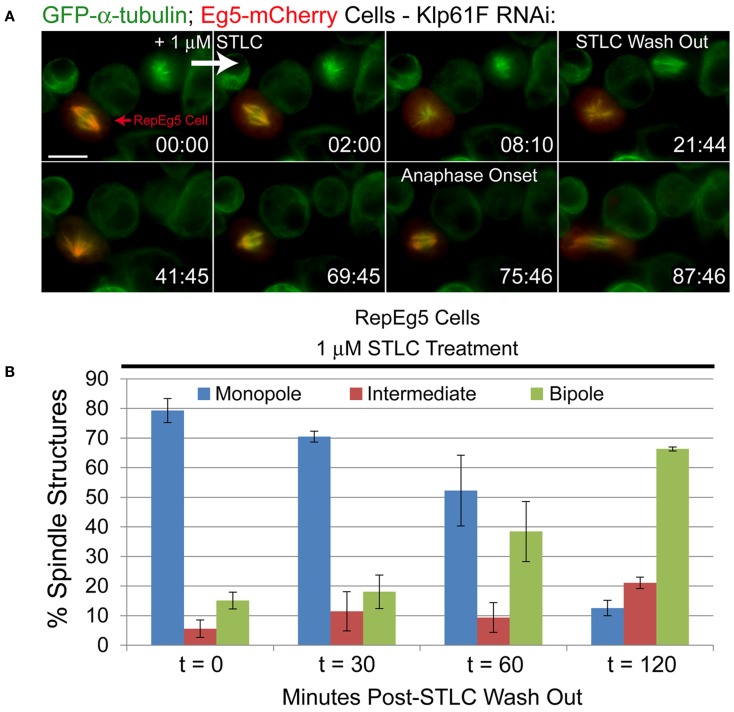
**Inhibition of Eg5 with STLC is reversible in RepEg5 cells**. **(A)** Still frames from a time-lapse of a RepEg5 cell before and after the addition of 1 μM STLC and following STLC wash out. STLC was added between 0:00 and 2:00 and washed out at 21:44. Addition of STLC causes the RepEg5 bipole to collapse into a monopole within 8 min. The RepEg5 monopole reorganizes into a bipole over ∼40 min following STLC wash out and undergoes a bipolar anaphase ∼54 min after removal of the drug. GFP-α-tubulin is shown in green and Eg5-mCherry is shown in red. **(B)** Quantification of spindle morphologies over time (in minutes) after STLC wash out (*t* = 0, *n* = 503 spindles; *t* = 30, *n* = 305; *t* = 60, *n* = 403; *t* = 120, *n* = 199). The percentage of monopoles decreases and the percentage of bipoles increases over time following removal of STLC. Error bars are SEM. Scale bar is 10 μm.

Since inhibition of Eg5 by STLC is reversible and the drug can be washed out of the cell types in which it is effective, the response of live RepEg5 cells to STLC treatment and drug wash out was examined (Figure 5A; Movie S6 in Supplementary Material). To do so, cells were treated for 30–60 min with 10 μM MG132 to delay mitotic cells in metaphase. Time-lapse imaging of metaphase cells was initiated and the starting media was exchanged with new media containing 1 μM STLC between acquisition of the first and second images in the time-lapse sequence. Spindle collapse and Eg5-mCherry spindle dissociation occurred rapidly and was completed within ∼10 min of adding the drug. Interestingly, unlike in mammalian cells where Eg5 activity is not required for maintaining spindle bipolarity once a bipolar spindle has assembled (46), Eg5 activity in RepEg5 cells is clearly required for both establishment and maintenance of spindle bipolarity. In this regard, spindles in RepEg5 cells behave more like those assembled in *Xenopus* egg extracts (47). After ∼20 min, the STLC-containing media was removed from the imaging chamber and replaced with fresh media lacking drug through three to four rounds of media exchange. In the example shown in Figure 5A, re-association of Eg5-mCherry with the spindle was evident within 20 min of removing STLC. The monopolar spindle reorganized into a bipolar configuration 50 min after the wash out and underwent a bipolar anaphase shortly thereafter (Figure 5A; Movie S6 in Supplementary Material).

To confirm the live-cell observations, spindle morphologies in fixed RepEg5 cells were quantified at various time points after wash out (Figure 5B). At *t*_0_, a vast majority of RepEg5 spindles were monopolar ( ∼80%) and only 13% of spindles were bipolar. The percentage of cells with bipolar spindles steadily increased over time following removal of STLC until 2 h after STLC wash out a significant majority (66%) of mitotic spindles in RepEg5 cells were bipolar (Figure 5B). Together, the fixed and live-cell data confirmed that STLC could in fact be washed out from *Drosophila* RepEg5 cells and that Eg5-mCherry localized and functioned properly after removal of the inhibitor.

### Robust error correction in RepEg5 cells requires aurora B kinase

By their very nature, monopolar spindles cannot establish proper bioriented kt-MT attachments. Syntelic kt-MT attachments, in which both sister kinetochores attach to the same spindle pole, are prevalent in monopoles (47). Erroneous attachments are not permanent; in fact, cells can correct improper kt-MT attachments, in large part, through activity of the centromere-enriched ABK (11, 21, 48). It was previously observed that the transition from monopole to bipole following Eg5 inhibitor wash out was accompanied by the conversion of syntelic attachments to bioriented attachments in an ABK-dependent manner (21).

**Figure 6:**
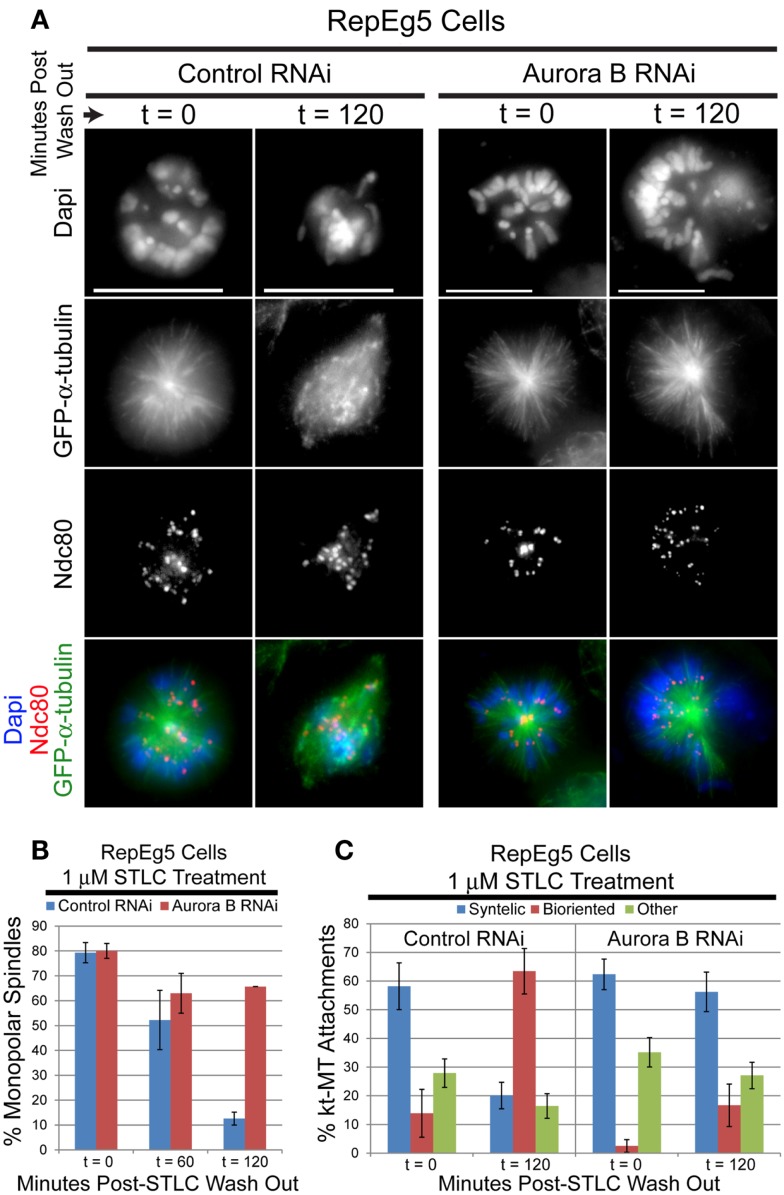
**Spindle bipolarization and error correction of kt-MT attachments following STLC wash out requires ABK**. **(A)** Representative maximum projection images of RepEg5 cells before and 2 hours after STLC wash out in the presence and absence of ABK. The cells were fixed and stained for α-tubulin (green), the kinetochore component Ndc80 (red), and DNA (blue). Two hours after removing STLC from control RNAi cells, a majority of spindles have converted from monopolar to bipolar with correction of most kt-MT attachments from syntelic to bioriented. In the absence of ABK, most spindles remain monopolar with a majority syntelic kt-MT attachments following STLC wash out. **(B)** Quantification of the percentage of monopolar spindles pre- and post-STLC wash out in RepEg5 cells with or without ABK. (Control RNAi, *t* = 0, *n* = 503 spindles; *t* = 60, *n* = 403; *t* = 120, *n* = 199; Aurora B RNAi, *t* = 0, *n* = 100 spindles; *t* = 60, *n* = 100; *t* = 120, *n* = 102). **(C)** Quantification of kt-MT attachments in RepEg5 cells in the presence and absence of ABK pre- and post-STLC wash out (Control RNAi, *t* = 0, *n* = 200 kinetochore pairs; *t* = 120, *n* = 193; Aurora B RNAi, *t* = 0, *n* = 210 kinetochore pairs; *t* = 120, *n* = 241). Error bars are SEM. Scale bars are 10 μm.

In order to validate the utility of RepEg5 cells in assaying error correction, the ability of STLC-treated RepEg5 cells to establish syntelic attachments and subsequently repair them following drug wash out was assessed in control and ABK depleted cells (Figures 6A,C). The outer kinetochore protein Ndc80 and microtubules were stained in RepEg5 cells to evaluate attachment state. Syntelic attachments were characterized by juxtaposed sister kinetochores attached to the same spindle pole while bioriented kinetochores were defined by the clear attachment of sister kinetochores to opposite spindle poles. Similar to other cell types, STLC-treatment in RepEg5 control RNAi cells resulted in elevated levels of syntelic attachments around monopolar spindles ( ∼60% of kt-MT attachments). In control cells, ∼90% of spindles were bipolar and a majority of syntelic attachments were converted to bioriented attachments (>60%) 2 h after washing out STLC. The outcome was dramatically different in ABK depleted cells. First, spindles failed to properly bipolarize (80% at *t*_0_ versus ∼65% at *t*_120_) following STLC wash out in the absence of ABK (Figure 6B). Second, syntelic attachments remained consistently high (>50%) for the duration of the STLC wash out experiment in the ABK knockdown cells as only 16% of kt-MT attachments were bioriented two hours after removal of STLC (Figure 6C). The quantification of attachments includes kinetochores from both bipolar ( ∼40%) and monopolar ( ∼60%) spindles. We included monopolar spindles in our analyses because, in our hands, ABK depletion from S2 cells leads to a preponderance of monopolar spindles. Thus, ABK activity is required for the conversion of monopolar to bipolar spindles and for efficient error correction after STLC wash out in RepEg5 cells.

## Discussion

In this study, a live-cell error correction assay has been successfully recapitulated in *Drosophila* S2 cells by functionally replacing *Drosophila* Klp61F with human Eg5. The error correction assay, which was initially developed in marsupial PtK_2_ cells (21), requires induction of erroneous attachments and the subsequent resolution of mal-oriented to bioriented kt-MT attachments in cells where kinesin-5 is reversibly inactivated with small molecule inhibitors. The fact that Ptk2 cells have relatively few large chromosomes and that the cells remain flat during mitosis makes them a model cell line for studying spindles, chromosomes, and kt-MT attachments; however, the availability and ease of application of genetic and molecular tools in PtK cells is quite limited. *Drosophila* S2 cells also have a relatively small number of chromosomes and can be flattened experimentally. Most importantly, there is an incredibly powerful range of experimental tools that can be applied to *Drosophila* cells including whole-genome RNAi screening. However, the error correction assay could not be applied in *Drosophila* cells due to the fact that available kinesin-5 inhibitors, including monastrol and STLC, do not inhibit Klp61F activity. This limitation has now been overcome.

### Studying the function of a human motor in a foreign cell type

At first glance, it may seem non-physiological to examine the function of human Eg5 in an alien cell type. However, the expression of Eg5-mCherry in *Drosophila* cells has been informative at multiple levels. The fact that human Eg5 rescues Klp61F depletion definitively demonstrates that the primary functions of kinesin-5 family members are conserved from fly to man. This is notable because of the fact that many *Drosophila* genes have rapidly diverged during evolution. In fact, numerous essential cell division regulators, kinetochore components in particular (49), have diverged to such a great extent that *Drosophila* homologs have been difficult to identify.

The ability of human Eg5 to localize and function normally in fly cells is also revealing about the nature of its regulation. For example, one of the most well-known regulators of Eg5 is the microtubule-associated protein TPX2 (37, 50, 51). The *Drosophila* homolog of TPX2 was only recently identified as Ssp1/Mei-38 (now called D-TPX2) (52). Interestingly, D-TPX2 reportedly lacks a highly conserved Eg5-binding domain, which has been shown to be required for proper Eg5 localization and function as well as normal spindle assembly in vertebrate cells (50, 51). There are several possible explanations for the discrepancy. First, D-TPX2 may indeed possess a highly diverged Eg5-binding motif that despite lacking any evident sequence similarity to the conserved domain still fulfills the function of interacting with kinesin-5 motors. Second, another *Drosophila* protein or proteins may fill the role of interacting with and regulating Eg5. Finally, intrinsic characteristics of human Eg5 in combination with organizational properties of the *Drosophila* spindle may negate the need for TPX2-style regulation of Eg5 in S2 cells.

The findings in RepEg5 cells are also relevant to current spindle assembly models. Balance of force/push-pull models of spindle assembly in vertebrate cells posit that Eg5 is directly antagonized by dynein to establish and maintain spindle bipolarity and spindle length (39–42). However, in *Drosophila* cells, the force that opposes Klp61F is predominantly generated by the minus-end directed kinesin-14 motor Ncd (29, 33, 34, 38, 43). Interestingly, spindle collapse in RepEg5 cells treated with STLC was largely dependent on Ncd activity. Thus, opposition of human Eg5 can be achieved in a dynein-independent manner. The data suggest that force-balance regulation of human Eg5 depends more on the existence of an opposing force than on the identity of the particular motor that is producing that force.

### Updating the first generation *Drosophila* cell-based error correction assay to v2.0

The work described here has taken an important first step in developing a potentially powerful new cell-based assay for studying error correction during mitosis. However, the first generation RepEg5 cell line has some limitations that could be overcome by creating RepEg5 version 2 (v2.0). First, RepEg5 requires the addition of CuSO_4_ to the cell media to induce Eg5-mCherry. It would be beneficial to develop cells with constitutive expression of Eg5-mCherry at levels comparable to endogenous Klp61F, which could be achieved by placing expression of Eg5-mCherry under control of the Klp61F promoter. Second, not every cell in the stable population used in this study expressed Eg5 – a property that proved useful in the current study by providing internal controls. However, the combination of a low baseline mitotic index in S2 cells with the fact that ∼25–50% of the cells expressed measurable levels of Eg5-mCherry made quantification time consuming. Thus, it would be useful to generate a clonal population of RepEg5 cells in which every cell expressed identical amounts of Eg5-mCherry at near-endogenous levels. Finally, depletion of Klp61F required a 2-day incubation of RepEg5 cells with exogenous dsRNA with homology to the Klp61F transcript. A stable RepEg5 cell line could be built that incorporates a constitutively expressed short hairpin RNA (shRNA) targeting Klp61F. Thus, RepEg5 v2.0 is envisioned as a clonal population of cells in which Klp61F has truly been replaced with human Eg5 by expressing Eg5-mCherry near-endogenous levels of Klp61F while constitutively blocking production of Klp61F. The RepEg5 v2.0 cell line would be amenable to high-throughput screening assays using STLC and available whole-genome RNAi libraries in combination with algorithms to score spindle morphology in a highly automated manner (24).

Failure to correct erroneous attachments results in CIN and aneuploidy. Aneuploidy in somatic cells has both tumorigenic and tumor protective properties (53) and CIN has been shown to promote tumor adaptation (54, 55). Thus, cell-based error correction screens have the potential to identify molecules that could be targeted by drugs in order to modulate CIN.

## Conflict of Interest Statement

The authors declare that the research was conducted in the absence of any commercial or financial relationships that could be construed as a potential conflict of interest.

## Supplementary Material

The Supplementary Material for this article can be found online at http://www.frontiersin.org/Molecular_and_Cellular_Oncology/10.3389/fonc.2013.00187/abstract

The Supplementary Material includes six videos and one table.

Supplementary Movie S1 **Montage of a two-color time-lapse of a *Drosophila melanogaster* S2 cell expressing GFP-α-tubulin (upper left; green in merge) and human Eg5-mCherry (upper right, red in merge) as it progresses through mitosis**. Lower left panel is the merged time-lapse. Scale bar is 10 μm.

Supplementary Movie S2 **Montage of a two-color time-lapse of a *Drosophila melanogaster* S2 cell expressing GFP-α-tubulin (upper left, green in merge) and human Eg5-mCherry (upper right, red in merge) as it progresses from metaphase through telophase**. Lower left panel is the merged time-lapse. Scale bar is 10 μm.

Supplementary Movie S3 **Time-lapse of a *Drosophila melanogaster* S2 cell expressing GFP-α-tubulin following addition of 1 μM STLC**. The STLC was added between frames 09:00 and 10:00. Note that the spindle does not collapse into a monopole. Scale bar is 10 μm.

Supplementary Movie S4 **Montage of a two-color time-lapse of a *Drosophila melanogaster* S2 cell expressing GFP-α-tubulin (upper left; green in merge) and human Eg5-mCherry (upper right, red in merge) following addition of 1 μM STLC between frames 0:00 and 02:27**. Note that the spindle does not collapse into a monopole despite the fact that spindle-associated levels of Eg5-mCherry drop following addition of the drug. Lower left panel is the merged time-lapse. Scale bar is 10 μm.

Supplementary Movie S5 **Montage of a two-color time-lapse of Klp61F-depleted cells**. The RepEg5 S2 cell expressing GFP-α-tubulin (upper left; green in merge) and human Eg5-mCherry (upper right, red in merge) is next to a cell that is not expressing Eg5-mCherry. Note that at the beginning of the movie the RepEg5 cell has a bipolar spindle while the nearby cell that is not expressing Eg5 has a monopolar spindle. The bipolar spindle in the RepEg5 cell rapidly collapses into a monopole following the addition of 10 μM STLC between frames 00:00 and 02:00 and remains a monopole for the duration of the experiment. Following addition of STLC, spindle-associated levels of Eg5-mCherry drops while cytoplasmic mCherry fluorescence increases. Lower left panel is the merged time-lapse. Scale bar is 10 μm.

Supplementary Movie S6 **Montage of a two-color time-lapse of Klp61F-depleted cells**. The RepEg5 S2 cell expressing GFP-α-tubulin (upper left; green in merge) and human Eg5-mCherry (upper right, red in merge) is next to a cell that is not expressing Eg5-mCherry. Note that at the beginning of the movie the RepEg5 cell has a bipolar spindle while a nearby mitotic cell that is not expressing Eg5 has a monopolar spindle. The bipolar spindle in the RepEg5 cell collapses into a monopole after addition of 1 μM STLC between frames 00:00 and 02:00. After washing out the STLC at 21:44 the monopole converts back to a bipole and undergoes anaphase starting at 75:46. Scale bar is 10 μm.

Supplementary Table S1 **The primers used in this study**. The underlined bases represent the T7 promoter sequence.

## Abbreviations

ABK, aurora B kinase; CIN, chromosomal instability; DMSO, dimethyl sulfoxide; GFP, green fluorescent protein; Klp61F, kinesin-like-protein 61F (*Drosophila* kinesin-5 motor); kt-MT, kinetochore-microtubule; Ncd, non-claret disjunction (*Drosophila* kinesin-14 motor); RepEg5 cells, replacement Eg5 cells; SEM, standard error of the mean; STLC, *S*-trityl-L-cysteine.
