# Supplementary material for: Development of a Drosophila Cell-Based Error Correction Assay
Source: Front Oncol. 2013 Jul 23;3:187. doi: 10.3389/fonc.2013.00187 (PMC3719216; doi:10.3389/fonc.2013.00187)
Supplement: Supplementary Table S1 — The primers used in this study. The underlined bases represent the T7 promoter sequence. [file 54754_Maresca_DataSheet1.PDF]

**Primer**	**Sequence**
KLP61F RNAi - Forward	TAATACGACTCACTATAGGGTATTTGCGCATTATTTTAAAA
KLP61F RNAi - Reverse	TAATACGACTCACTATAGGGATATTGATCAATTGAAAC
Ncd RNAi - Forward	TAATACGACTCACTATAGGGATGGAATCCCGGCTACCGAA
Ncd RNAi - Reverse	TAATACGACTCACTATAGGGGTGGAAGCGGGCCTTGAAGT
Aurora B RNAi - Forward	TAATACGACTCACTATAGGGATGACGCTTTCCCGCGCGAA
Aurora B RNAi - Reverse	TAATACGACTCACTATAGGGTTGAGGGCATTCGCCACTTG
